# Changes in the lodging resistance of winter wheat from 1950s to the 2020s in Henan Province of China

**DOI:** 10.1186/s12870-023-04452-z

**Published:** 2023-09-20

**Authors:** Yang Wang, Yonghui Pan, Fulin Zhao, Xiangping Meng, Qun Li, Yudfang Huang, Youliang Ye

**Affiliations:** 1 Agricultural Green Development Engineering Technology Research Center, College of Resources and Environment, Henan Agricultural University, Zhengzhou, 450002 China; 2 College of Life Sciences and Oceanography, Shenzhen University, Shenzhen, 518060 China

**Keywords:** Wheat, Lodging resistance, Stem breaking strength, Genetic improvement, Nitrogen fertilizer regimen

## Abstract

**Background:**

Lodging is a major factor contributing to yield loss and constraining the mechanical harvesting of wheat crops. Genetic improvement through breeding effectively reduced the lodging and improved the grain yield, however, the physiological mechanisms involved in providing resistance to lodging are different in the breeding stage and are not clearly understood. The purpose of this study was to compare the differences in the lodging resistance (LR) of the wheat varieties released during the different decades and to explore the effect of the application of nitrogen (N) fertilizer on the plasticity of LR.

**Results:**

A field study was conducted during the cultivation seasons of 2019–2020 and 2020–2021, in soil supplemented with three N levels: N_0_ (0 kg ha^–1^), N_180_ (200 kg ha^–1^), and N_360_ (360 kg ha^–1^) using eight varieties of wheat released for commercial cultivation from 1950 to date. The results obtained showed that genetic improvement had significantly enhanced the LR and grain yield in wheat. In the first breeding stage (from 1950 to 1980s) the lodging resistant index increased by 15.0%, which was primarily attributed to a reduced plant height and increased contents of cellulose, Si, and Zn. In the second breeding stage (the 1990s–2020s) it increased by 172.8%, which was mainly attributed to an increase in the stem diameter, wall thickness, and the contents of K, Ca, Fe, Mn, and Cu. The application of N fertilizer improved the grain yield but reduced the LR in wheat. This was mainly due to an increase in plant height resulting in an elevation of the plant center of gravity, a decrease in the contents of cellulose, and a reduction in the area of large-sized vascular bundles in the stems, even if N supplementation increased the concentrations of K, Ca, and Si.

**Conclusion:**

Although breeding strategies improved the stem strength, the trade-off between the grain yield and LR was more significantly influenced by the addition of N. Overcoming this peculiar situation will serve as a breakthrough in improving the seed yield in wheat crops in the future.

## Introduction

Wheat (*Triticum aestivum* L.), is one of the world’s most widely cultivated crops and plays an important role in ensuring national food security [1]. Global wheat productivity has been increasing gradually due to the continuous development of technology. However, wheat cultivation still faces a myriad of challenges such as lodging risks that threaten crop yield [2]. Wheat lodging is mainly caused by extreme and destructive weather events, which have been recently occurring more frequently. Stem lodging in wheat is a major agronomic problem that has far-reaching economic consequences by limiting the yields of cereals in both developed and developing countries [3, 4]. It is estimated that lodging leads to a loss of up to 60%–80% in the yield of wheat crops due to a reduced photosynthetic capacity resulting due to the destruction of the canopy structures [5].

Stem lodging usually occurs at the basal second internode, and it is correlated with plant height and stem strength [6]. For overcoming this problem, the wheat breeders have improved the lodging resistance (LR) through the technique of stem dwarfing. The “Green Revolution,” which saw an enhanced application of dwarf-plant-based breeding methods, has contributed to a sharp reduction in the lodging risk and an increase in the average yield of wheat [7]. Recent progress in Green Revolution has been made through the identification of the *Reduced height* (*Rht*) genes, including both the gibberellic acid (GA)-sensitive and GA-insensitive loci [8, 9]. In addition to reducing the plant height (PH), certain *Rht* loci also affect the architecture of the inflorescence and thus may influence grain yield (GY) [10, 11]. Quantitative trait loci (QTL) have also been reported for stem strength, culm wall thickness, pith diameter and stem diameter, which are associated with wheat lodging resistance [4, 12]. A single solid stem QTL was identified on chromosome 3BL, which also contributes to lodging resistance [13]. A common genomic region affecting overall stem strength, including internode material strength, internode diameter, and internode wall width, is located in the interval 278–287 cM of chromosome 3B [14]. Many QTL for plant height have also been identified with most of them located close to those reported *Rht* genes [15, 16]. The results of a comparative test between the winter wheat cultivars developed in the UK since the twentieth century revealed that, the PH was reduced and the GY was enhanced through genetic improvement [17]. Similarly, the PH was observed to gradually decrease with the application of breeding through the years since the 1960s in China [18].

The enhancement in the GY is hampered by the commonly occurring problem of crop lodging, which is perhaps underestimated and is constantly aggravating as the yield increases [19]. Improving the LR in crops by reducing the PH is limited only up to a certain extent in the currently used methods of crop production [20]. Several studies have suggested that the yield is reduced markedly when the PH is restricted to below a certain level using dwarfing genes [21, 22]. An unscientific approach to dwarfing can limit the GY, hence other methods of improving the LR need to be considered. One such method is the modification of the structure of the stem tissues [23]. Previous studies on the microstructure of stems using the cross-sections of the basal regions of the stems revealed that the tissues were highly lignified and the stems organized with a significant number of large- and small-sized vascular bundles of increased area thus improving the mechanical strength in the lodging resistant wheat cultivars [13]. Fiber which mainly consists of cellulose, hemicelluloses, and lignin plays an important role in the mechanical support of the cell wall [24]. Among these components, cellulose is the main one and has contributed to maintaining culm mechanical strength. Several qualitative characteristics such as crystallinity contribute to the strength of cellulose fibers [25]. Furthermore, the levels of certain mineral nutrients such as K and Si were closely correlated to LR [26, 27]. The anatomical structure and the mineral nutrient levels in the stem have significant impacts on the LR; however, the causes behind these variations which occur during the breeding process are currently ambiguous.

Undoubtedly, the genetic improvement of crops and the addition of chemical-based fertilizers especially nitrogen (N) fertilizer are the two most important factors affecting agricultural output [28]. Previous studies have revealed the mechanisms behind the influence of N in improving the yield and quality of crops from the perspective of agronomic traits, photosynthesis and physiology, ecological effects, and nutrient absorption and transport [29, 30]. Intensive methods of crop management, such as excessive application of N fertilizers and a high planting density enhanced the susceptibility of plants to lodging, which was mainly due to an increased stem length, decreased stem diameter, and reduced cell-wall thickness (WT) that may diminish the parameters of flexural rigidity and breaking resistance which indicate physical strength [31]. Additionally, N luxury significantly reduced the generation of H, G, and S monomers of the lignin (related to stem hardness) as well as its total content in crop stem [32]. Reduced breaking strength and higher lodging index in wheat under higher N were associated with reduced cellulose contents in the culm [33]. Some microelements (Si, Ca, Mg, Fe, Zn, Cu, and Mn) in the culm can also be affected by N application, resulting in lower breaking strength and higher lodging index in rice plants, in particular breaking strength of culm which plays a key role in lodging index [34].

The production of biomass and GY were concomitantly reduced with the decreasing application of N [35]. Achieving a simultaneous increase in both the GY and LR seems to be difficult in wheat. However, studies have been conducted to understand the trade-off between the two and assess the possibility of managing the lodging risk without an associated reduction in yield [13]. In this study, the differences in the GY and LR in selected wheat varieties obtained through genetic improvement under varying regimens of N fertilizer were undertaken. The main objective was to explore the effect of agronomic characters and physiological mechanisms (in terms of stem anatomical structure, mineral element and cellulose) on the trait of LR caused due to genetic improvement and N regimes. The findings can help in understanding the adaptability of genetically improved crops to N fertilizer and provide a scientific reference that can be accessed before initiating crop breeding for the efficient utilization of resources in the future.

## Results

### Improvement of yield and yield-related parameters of the varieties developed over the decades

**Fig. 1:**
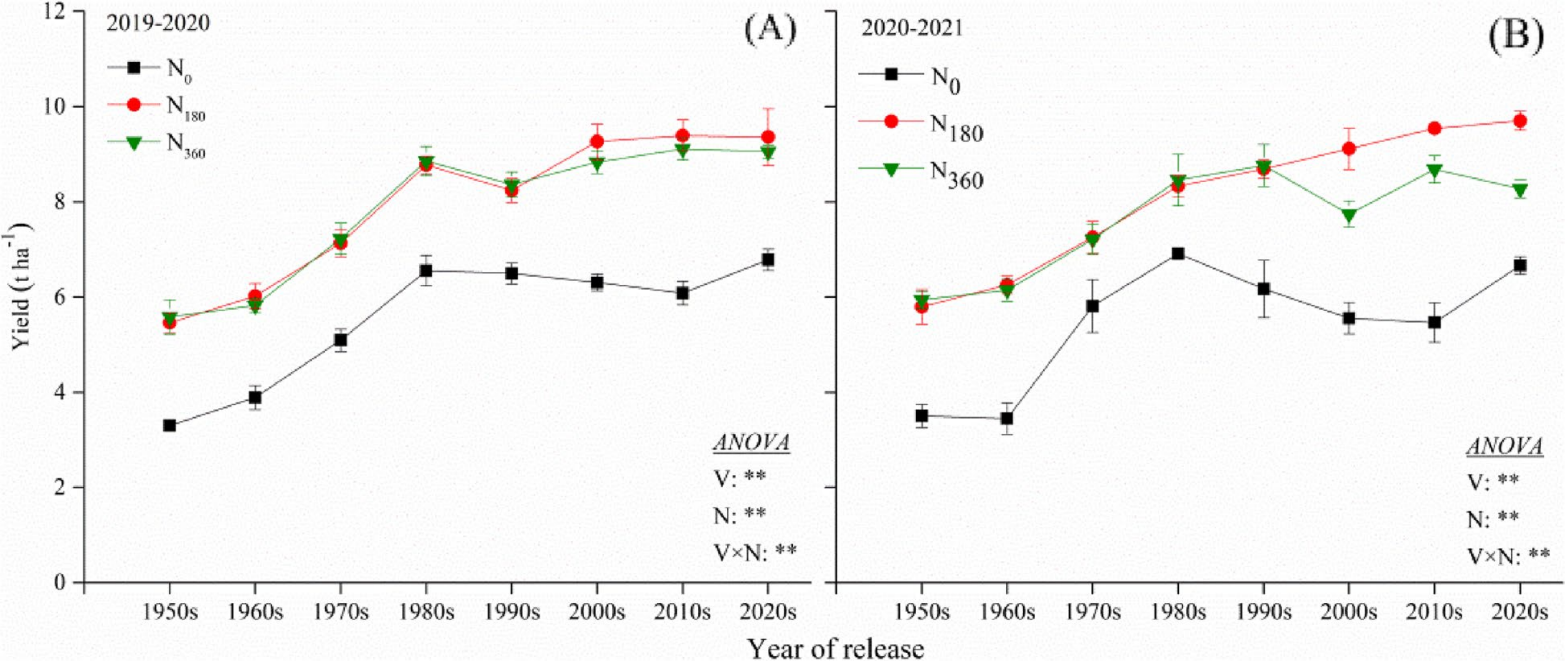
Yield of the different wheat varieties during 2019–2020 (**A**) and 2020–2021 (**B**). ** indicate that the yield is significantly influced by the variety, N rate and their interactions at 0.01 levels

Two-way ANOVAs revealed that the grain yield was markedly affected by the variety (V), N rate (N) and their interaction effect (V × N) during 2019–2020 and 2020–2021 (Fig. 1). Compared with the cultivar developed in the 1950s, the yield of the cultivars developed in the 1960s, 1970s, 1980s, 1990s, 2000s, 2010s, and 2020s demonstrated an increase of 9.7%, 35.5%, 68.5%, 60.9%, 70%, 71.3%, and 75.6% during the experimental period of 2019–2020 respectively; and of 3.9%, 33%, 55.4%, 54.9%, 47.1%, 55.4%, and 61.6% during 2020–2021, respectively. The mean decadal rate of increase in the yield of the cultivars from the 1950s–1980s was 1.81 kg ha^−1^ decade^−1^ during the experimental period of 2019–2020, and 1.56 kg ha^−1^ decade^−1^ during 2020–2021. The mean decadal rate of increase in the yield of the cultivars from the 1990s–2020s was 3.32 kg ha^−1^ decade^−1^ during 2019–2020, and 2.78 kg ha^−1^ decade^−1^ during 2020–2021. The application of N fertilizer significantly increased the GY in all eight cultivars, but excessive input of N did not further improve the GY but inhibited it. In comparison with the N_0_ treatment, the GY at N_180_ increased by 65.1% (the 1950s), 54.7% (1960s), 40% (1970s), 33.8% (1980s), 26.8% (1990s), 46.9% (2000s), 54.3% (2010s), and 38% (2020s) during the experimental season of 2019–2020; and by 65% (1950s), 81.6% (1960s), 24.7% (1970s), 20.6% (1980s), 40.8% (1990s), 64% (2000s), 74.5% (2010s), and 45.5% (2020s) during the experimental season of 2020–2021. At N_360_, the GY of all the cultivars improved less rapidly or even declined when compared to that at N_180_.

Two-way ANOVAs revealed that the number of panicles per m^2^ (NPM), the number of grains per panicle (NGP) and 1000-grain weight (TGW) were markedly affected by variety (V) and N rate (N). Also, the effects of V and N interactions (V × N) on NPP and NGP were highly significant (Table 1). NPM, NGP and TGW are crucial indicative parameters indicating the yield of a crop. The NGP and TGW increased with era processing, whereas the NPM decreased obviously. The NPM in all the cultivars enhanced significantly with the increasing input of N (Table 2). Except in the case of the cultivars from the 1950s and 1970s, the NGP was markedly elevated at N_180_ over the N_0_ treatment but then reduced at the N_360_ treatment. The TGW was marginally affected by the addition of N fertilizer, the order being N_180_ > N_360_ > N_0_.

**Table 1:** Yield parameters in the wheat varieties after growth under different N regimens during the experimental seasons of 2019–2020 and 2020–2021. The least significant difference (LSD) test was performed to compare the mean values of the various treatments. The lowercase letters after the numbers in the same column indicate that the differences between the three N levels for the same variety were significant at the level of 0.05, and the capital letters after the numbers in the same column indicate that the differences between the eight varieties were significant at the level of 0.05. ** indicate that the yield components were significantly influenced by variety, N rate, and their interactions at 0.01 levels, and ns indicates ‘not significant’

Variety	N treatment (kg ha^−1^)	Number of panicles (m^−2^)		Number of grains per panicle		1000-grain weight (g)	
		2019–20	2020–21	2019–20	2020–21	2019–20	2020–21
ND2419 (1950s)	0	377.7c	401.0c	32.7a	32.3a	35.1b	36.2c
	180	527.0b	558.3b	30.6a	30.7ab	38.3a	39.8a
	360	630.0a	666.3a	27.5b	28.1b	35.9ab	38.1b
	Mean	511.6A	541.9A	30.2E	30.4G	36.4E	38.0E
BJ8 (1960s)	0	343.7b	381.0c	36.1b	33.7a	31.7a	29.0c
	180	491.7a	536.7b	42.4a	35.8a	33.2a	34.2a
	360	526.0a	610.7a	39.1ab	35.5a	32.3a	31.5b
	Mean	453.8BC	509.4B	36.8C	35.0F	32.4F	31.6F
ZY1 (1970s)	0	334.7b	320.3b	38.9a	38.0a	42.1a	44.0a
	180	530.3a	544.0a	36.6b	34.9b	43.1a	43.8a
	360	562.0a	579.0a	35.1c	36.1ab	42.0a	39.2b
	Mean	475.7B	481.1C	39.2D	36.3F	42.4D	42.3C
XY4 (1980s)	0	364.0c	358.7c	39.8ab	40.0a	42.8b	43.1b
	180	424.7b	421.3b	41.4a	40.5a	46.6a	46.3a
	360	506.0a	509.0a	38.5b	37.3b	43.0b	42.1b
	Mean	431.6CD	429.7D	39.9C	39.2E	44.1C	43.8B
BN3217 (1990s)	0	369.7c	379.3c	42.5a	40.9b	42.1a	42.3a
	180	486.7b	507.0a	43.9a	44.2a	42.2a	41.4ab
	360	531.3a	551.3a	42.7a	41.7b	40.1a	39.2b
	Mean	462.6BC	479.2C	43.0B	42.2D	41.5D	41D
YM2 (2000s)	0	339.3b	326.0c	40.8a	41.8c	45.4a	44.2a
	180	440.0a	441.0b	45.0a	46.3a	45.7a	45.6a
	360	470.3a	477.7a	43.5a	43.7b	44.1a	42.1b
	Mean	416.6D	414.9D	43.1B	44.0C	45.1C	44.0B
BN207 (2010s)	0	273.3b	254.7b	43.7b	44.7b	46.9a	46.6b
	180	394.0a	376.0a	47.5a	51.4a	47.7a	48.4a
	360	405.7a	402.0a	45.7ab	48.3ab	46.8a	47.0b
	Mean	357.7E	344.2F	45.6A	48.2A	47.1B	47.3A
BN4199 (2020s)	0	296.3b	302.3b	43.8a	44.4b	47.4a	46.3b
	180	414.0a	420.0a	46.1a	49.5a	50.2a	50.2a
	360	434.0a	423.0a	45.9a	45.9b	48.8a	48.6a
	Mean	381.4E	381.8E	45.2A	46.6B	48.8A	48.4A
V		**	**	**	**	**	**
N		**	**	**	**	**	**
V × N		**	**	**	**	ns	ns

**Table 2:** Mechanical tissue thickness, wall thickness, cellulose content, and anatomical structure of the stems of the wheat varieties after growth under different N regimens. The number of small vascular bundles, NSVB; the number of large vascular bundles, NLVB; the area of small vascular bundles (μm^2^), ASVB; the area of large vascular bundles (μm^2^), ALVB; cellulose content, CC; mechanical layer thickness (μm), MLT; wall thickness (μm), WT. The least significant difference (LSD) test was performed to compare the mean values of the various treatments. The lowercase letters after the numbers in the same column indicate that the differences between the three N levels for the same variety were significant at the level of 0.05, and the capital letters after the numbers in the same column indicate that the differences between the eight varieties were significant at the level of 0.05. ** indicate that the anatomic parameters were significantly influenced by variety, N rate, and their interactions at 0.01 levels, and ns indicates ‘not significant’

Variety	N treatment (kg ha^−1^)	NSVB		NLVB		A SVB (× 10^4^ μm^2^)		ALVB (× 10^4^ μm^2^)		CC (%)		MLT (μm)		WT (μm)	
		2019–20	2020–21	2019–20	2020–21	2019–20	2020–21	2019–20	2020–21	2019–20	2020–21	2019–20	2020–21	2019–20	2020–21
ND2419 (1950s)	N0	22.0b	23.0b	37.0a	36b	11.2b	10.7b	83.2a	84.3a	40.4a	38.3a	75.6c	82.2b	126.5a	110.9a
	N180	27.3ab	27.7a	38.0a	39.7a	13.1a	13.5a	73.0b	83.9a	39.7a	37.4ab	91.1b	89.3b	99.8b	83.4b
	N360	25.3a	26.0a	40.3a	40a	14.7a	13.8a	73.5ab	81.2a	38.5a	36.0b	108.7a	115.1a	93.6b	92.9b
	Mean	24.9BC	25.6C	38.4D	38.6D	13.0A	12.7A	76.5E	83.1EF	39.5AB	37.3B	91.8EF	95.5CD	106.6DE	95.7E
BJ8 (1960s)	N0	15.7b	15.7c	38.7a	37.7a	7.7c	6.2c	97.1a	83.8a	39.0a	42.2a	79.6a	76.1b	120.2a	107.8a
	N180	21.3a	22.3b	35.7a	34b	9.1b	8b	83.2b	81.5ab	38.5a	40.3a	87.2a	81.8ab	111.5a	94.5b
	N360	24.7a	25.7a	38.3a	38.3a	12.2a	11.8a	80.3b	72b	36.1b	37.9b	91.4a	86.4a	96.4b	87.2c
	Mean	20.6EF	21.2E	37.5D	36.7D	9.7F	8.6F	86.9D	79.1F	37.9BC	40.2A	86.1G	81.4E	109.4D	96.6E
ZY1 (1970s)	N0	27.7a	27.0b	34a	36a	11.3b	11.7a	71.3a	89.9a	41.1a	41.0a	83.7b	82.4b	124.0a	135.2a
	N180	28.3a	29.0a	37.7a	38.3a	10.8b	11.9a	69.4a	84.3ab	40.8a	39.7a	90.3b	86.8b	87.7b	971b
	N360	28.7a	29.0a	39.7a	41a	13.9a	12.2a	68.8a	81.9b	38.4b	36.3b	108.5a	109.6a	91.9b	103.9b
	Mean	28.2A	28.3B	37.1D	38.4D	12.0B	12.0B	69.8F	85.3EF	40.1A	39.0AB	94.2DE	92.9D	101.2E	112.1D
XY4 (1980s)	N0	19.3b	20.7b	39.3a	40.7b	10.6a	5.7c	86.3a	99.2a	42.4a	42.0a	74.3c	68.6b	115.1a	1173.1a
	N180	24.7a	22.7ab	42a	52.7a	11.4a	10.9a	81.9a	91.8a	40.2ab	39.6ab	87.9b	88.3a	107.6a	92.7b
	N360	25.3a	24.7a	41a	46.3ab	11.0a	8.8b	66.7b	80b	39.0b	36.3b	101.5a	92.2a	86.4b	87.3b
	Mean	23.1CD	22.7DE	40.8C	46.6B	11.0CDE	8.4F	78.3E	90.3DE	40.5A	39.3AB	87.9FG	83.0E	103.0DE	99.1E
BN3217 (1990s)	N0	16.7b	15.7b	37.3b	38.7b	7.9c	7.4b	96.8a	100.0a	41.0a	40.2a	89c	96.6b	119.7a	114.4a
	N180	17.3b	16.3b	44.3a	48a	11.7b	8.1b	93.3a	95.7a	39.6ab	39.5a	119.7b	102.5b	84.5b	96.3b
	N360	26a	23.3a	43.3a	42.3b	15.3a	13.3a	92.8a	89.7a	38.2b	35.4b	132.6a	138.1a	77.1b	85.1b
	Mean	20F	18.4F	41.7C	43BC	11.7BC	9.6E	94.3C	95.1CD	39.6AB	38.4AB	114.0B	112.4B	93.8F	98.6E
YM2 (2000s)	N0	22.7b	24.7b	40.7b	40.7a	8.2c	8.1b	111.8a	106.5a	42.8a	41.1a	98.3b	86.2b	143.9a	141.0a
	N180	27a	27.3a	43.7ab	45a	10.7b	8.8b	109.7a	101.4a	40.5b	39.7ab	103.8b	97.9a	136.4a	137.3a
	N360	27.7a	26.3ab	46a	42.3a	12.3a	11.5a	105.2a	99.7a	37.1c	36.5b	119.0a	101.1a	112.3b	120.5b
	Mean	25.8B	26.1C	43.4B	42.7C	10.4EF	9.4E	108.9A	102.6B	40.1A	39.1AB	107.0C	95.1CD	130.9C	132.9C
BN207 (2010s)	N0	17c	17.7c	45b	48.7b	8.1c	8.6b	117.4a	125.4a	37.5a	36.0a	112.8c	116.2c	181.8a	202.8a
	N180	22.7b	23b	53a	59a	11.1b	9.7b	110.7a	118a	36.8a	34.9ab	122.1b	128.7b	158.12b	180.5b
	N360	27a	28.3a	51.3a	52b	12.7a	12.5a	106.6a	109.1a	34.2b	33.6b	133.3a	141.2a	138.4c	121.7c
	Mean	22.2DE	23D	49.8A	53.2A	10.7DE	10.3D	111.5A	117.5A	36.1C	34.9C	123.0A	128.7A	159.5A	170.1A
BN4199 (2020s)	N0	23b	23.7b	41b	42b	10.3b	9.7b	104.1a	106.4a	39.1a	40.0a	90.4a	87.8b	173.0a	185.5a
	N180	32.7a	34.0a	43.7a	43b	18.8a	10.7b	103.2a	101a	37.5ab	38.5a	98.0a	100.8a	147.1b	154.9ab
	N360	31.7a	32.3a	45.7a	49a	11.4ab	13.6a	100.7a	96.4a	35.1b	34.9b	105.2a	105.2a	129.4c	128.17b
	Mean	29.1A	30.0A	43.5B	44.6BC	11.5BCD	11.4C	102.7B	101.2BC	37.2C	37.8B	97.9D	97.9C	149.8B	156.2B
V		**	**	**	**	**	**	**	**	**	**	**	**	**	**
N		**	**	**	**	**	**	**	**	**	**	**	**	**	**
V × N		**	**	ns	ns	ns	ns	ns	ns	ns	ns	**	**	**	**

### Lodging rate and lodging resistance index (LRI)

**Fig. 2:**
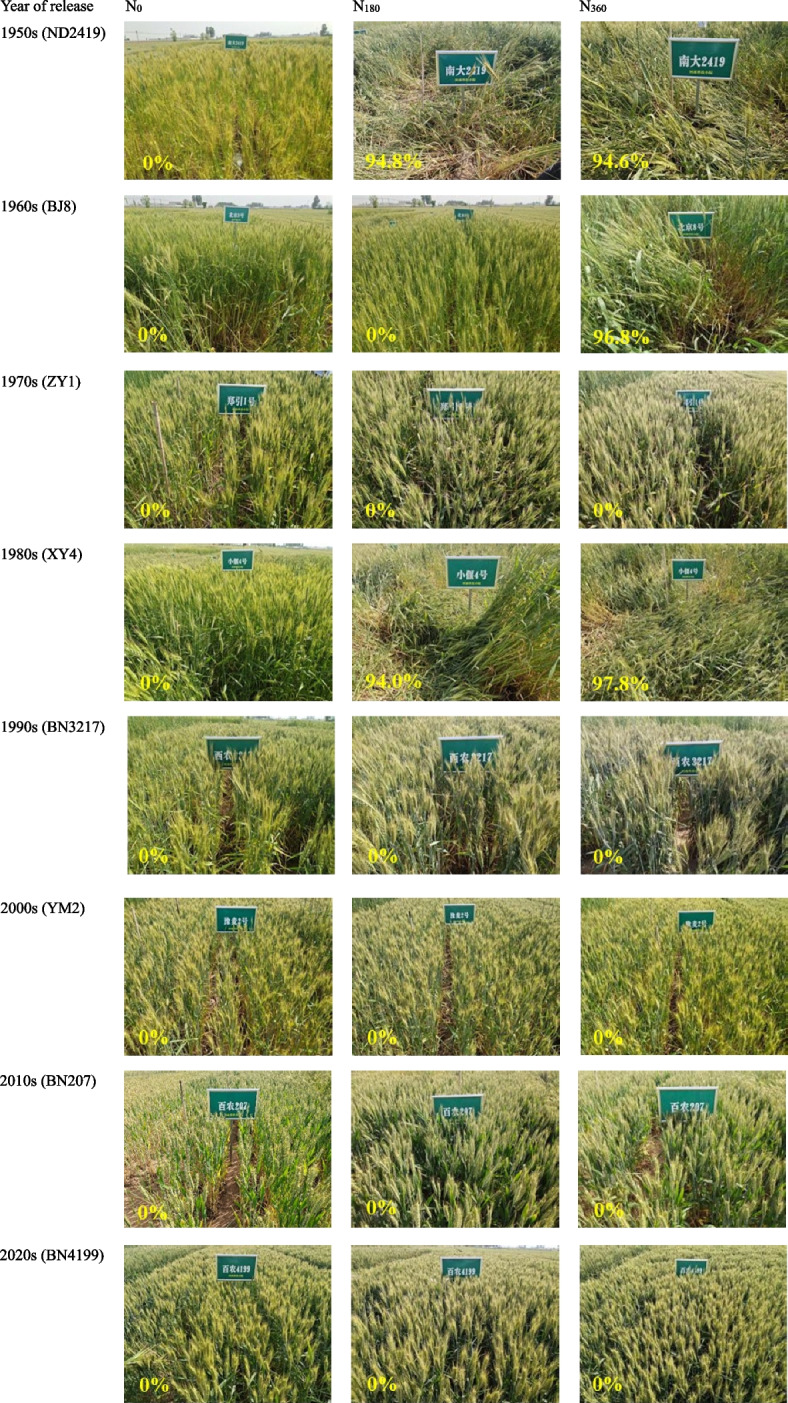
Pictures of the wheat field taken at the grain-filling stage during 2020–2021. Yellow values represent the two-year average wheat lodging incidence

**Fig. 3:**
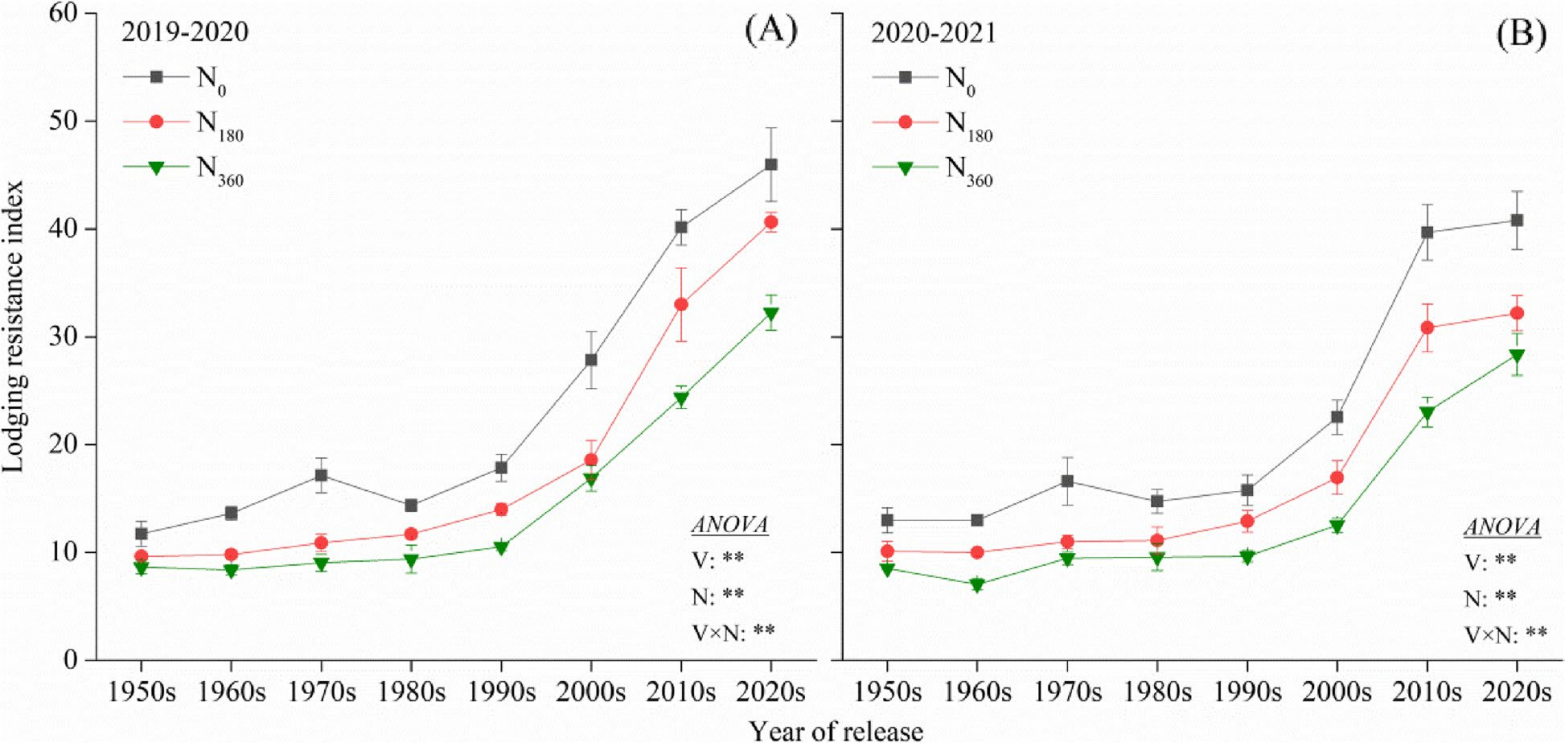
Lodging resistance index (LRI) of the different wheat cultivars at the grain filling stage during the experimental seasons of 2019–2020 (**A**) and 2020–2021 (**B**). ** indicate that the LRI is significantly influced by the variety, N rate and their interactions at 0.01 levels

During both the experimental seasons, natural lodging was observed in the three cultivars- ND2419, BJ8, and XY4 but not in others (Fig. 2). The plants of these three cultivars did not demonstrate lodging at N_0_ but at the N_360_. This suggested that the LRI increased through genetic improvement; the LRI of the cultivars from the 2000s, 2010s, and 2020s was 138.6% (2019–2020) and 27.4% (2020–2021) higher than those from the 1950s–1990s (Fig. 3). Furthermore, the LRI was observed to be negatively correlated with the amount of N fertilizer.

### PH, PCG, and the CC of the stems

**Fig. 4:**
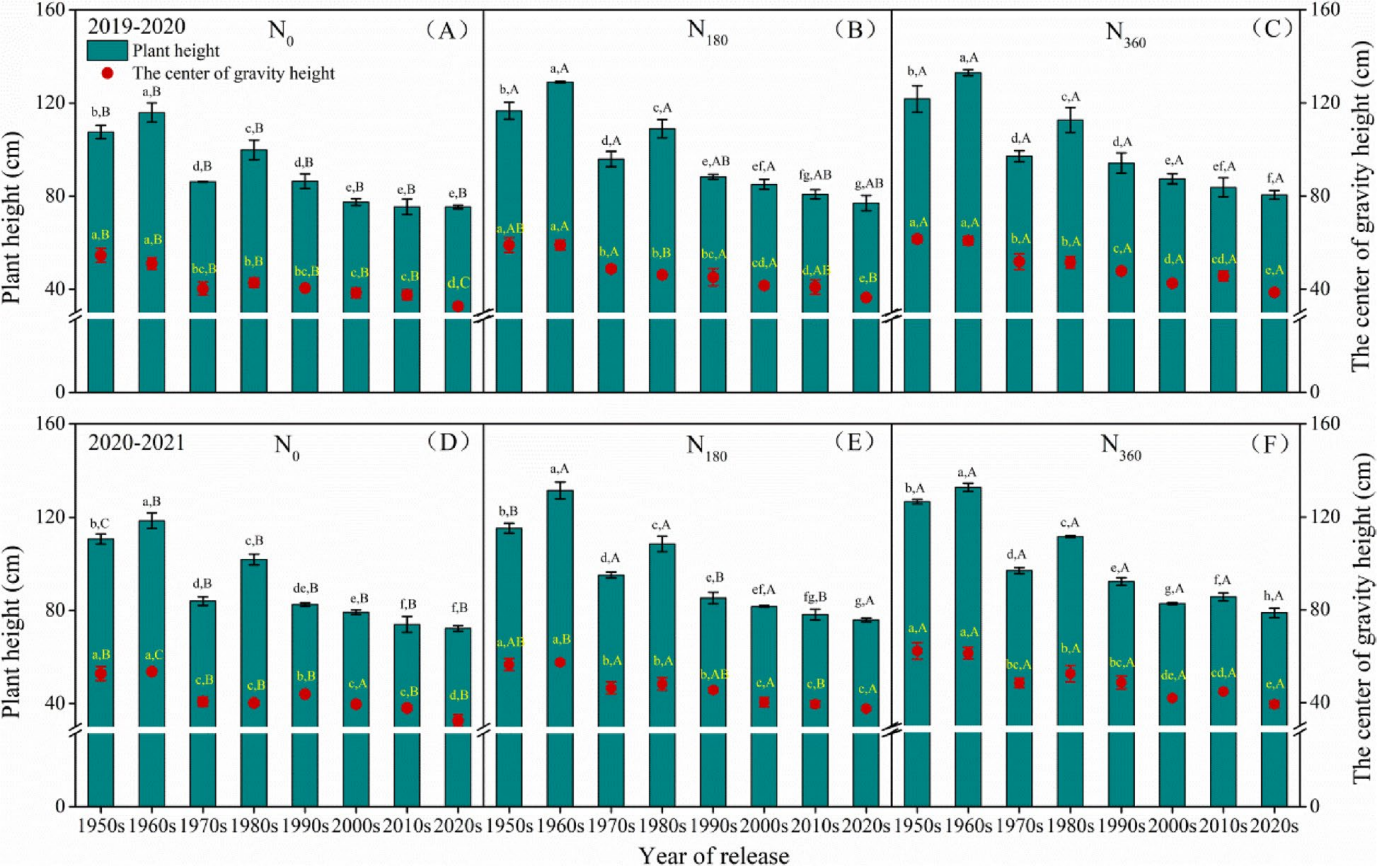
Plant height and plant center of gravity of the wheat varieties at different ages under the three N regimens during 2019–2020 (**A**–**C**) and 2020–2021 (**D**–**F**). The different lowercase letters indicate significant differences (*p* < 0.05) among different varieties at the same N condition. The different uppercase letters indicate significant differences (*p* < 0.05) among different N rates for the same variety

The PH and PCG gradually decreased with the ear processing (Fig. 4A–F). The application of N fertilizer increased the PH and elevated the PCG in all the cultivars. Compared to the control, the increase in the height of the plants of the cultivars from the1950s, 1960s, 1970s, 1980s, 1990s, 2000s, 2010s, and 2020s at N_180_ was 8.5%, 11.2%, 11.4%, 9.1%, 2.2%, 9.8%, 7.2%, and 2.3% during 2019–2020, respectively; and was 4.2%, 10.9%, 13.3%, 6.5%, 3.3%, 3.2%, 5.7%, and 5.0% during 2020–2021, respectively (Fig. 4B, E). Similarly, in comparison to N_0_, the elevation in the PCG at N_180_ was enhanced by 8%, 15.5%, 21.1%, 7.9%, 11.6%, 8.2%, 8.9%, and 11.4% during 2019–2020, respectively; and by 7.4%, 7.3%, 14.2%, 20.3%, 4%, 2.3%, 4.5%, and 15.7% during 2020–2021. It is worth noting that the cultivar from the 1970s had the highest increase in the PH and elevation of the PCG with the addition of N fertilizer. This effect was lower in the N_360_ vs. N_180_ group than in the N_180_ vs. N_0_ group (Fig. 4C, F). At N_180_, the maximum increase in the PH and elevation in the PCG were 6.6% and 11.5% during 2019–2020; and 9.8% and 12.3% during 2020–2021, respectively.

In the cultivars from the 1950s–1990s, the CC did not change significantly during 2019–2020, but it began to decline in those from the 2010s (Table 2). The average CC of the cultivars from the 1950s–2000s was 7.0% (2019–2020) and 8.0% (2020–2021) higher than those from the 2010s–2020s. The CC demonstrated a reciprocal relation with N. Compared to N_0_ treatments, the cellulose content in the cultivars from the 1950s, 1960s, 1970s, 1980s, 1990s, 2000s, 2010s, and 2020s at N_360_ was reduced by 4.8%, 7.3%, 6.5%, 7.9%, 7.0%, 13.1%, 8.8%, 10.2% during 2019–2020, respectively; and enhanced by 6.1%, 10.3%, 11.3%, 13.6%, 12.1%, 11.1%, 6.6%, and 12.8% during 2020–2021, respectively.

### SBS and microstructure of the second internode of the stems

**Fig. 5:**
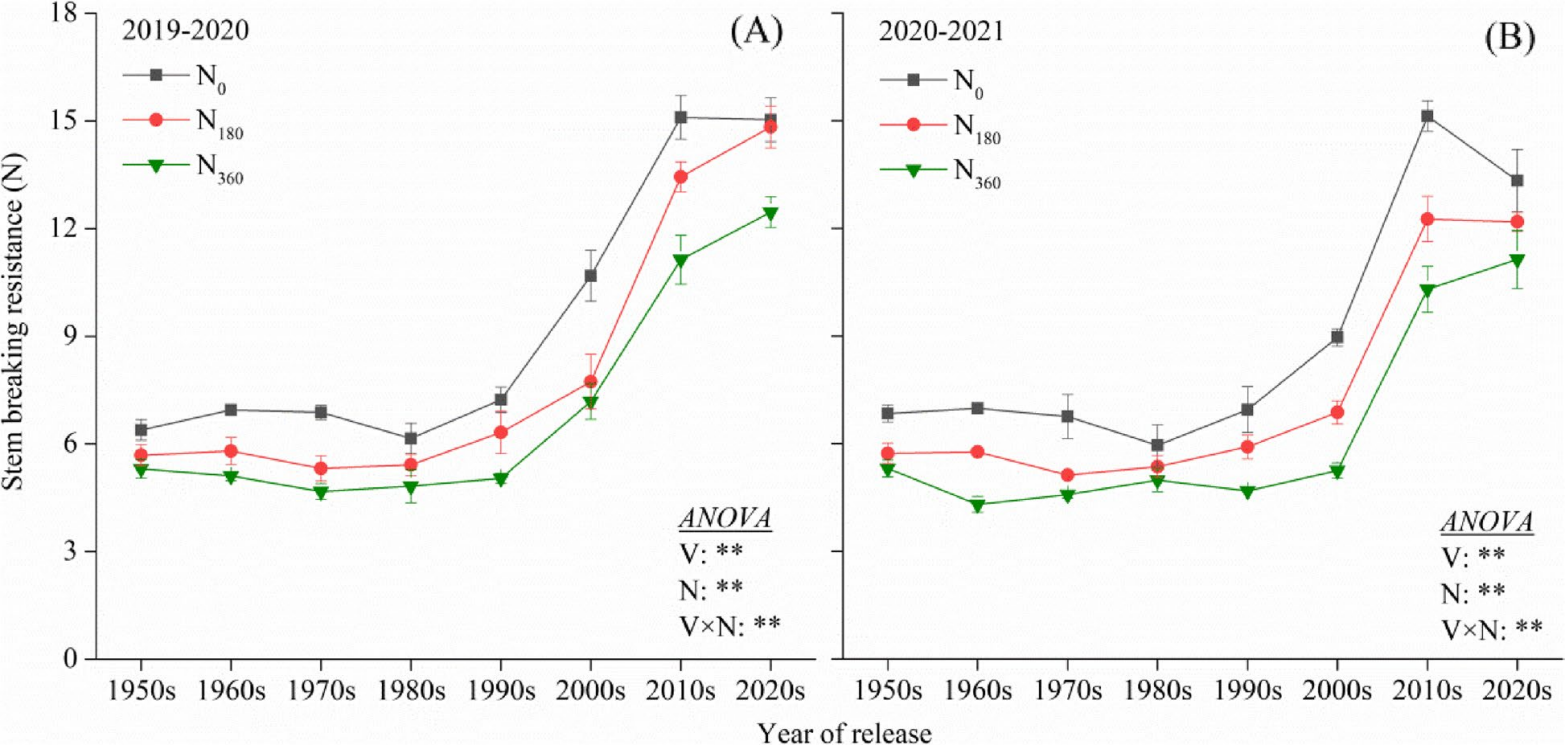
Stem breaking strength (SBS) of the second internode of the different wheat varieties during 2019–2020 (**A**) and 2020–2021 (**B**). ** indicate that the SBS is significantly influced by the variety, N rate and their interactions at 0.01 levels

Two-way ANOVAs revealed that the SBS and microstructure were markedly affected by the variety (V) and N rate (N), and there was interaction effect on the SBS, NSVB, MLT and WT. No significant changes in the SBS among the wheat cultivars from the 1950s–1980s were observed in both the experimental seasons (Fig. 5). However, in comparison to these, the SBS of the cultivars from the 1990s–2020s substantially increased by 45.7% (2019–2020) and 40.1% (2020–2021) on an average, respectively. Application of N fertilizer resulted in a reduction of the SBS, the mean SBS at N_180_ reduced by 13.3% and 16.5% in comparison to that at N_0_; and by 13.7% and 14.6% at N_360_ in comparison with that at N_180_.

The mean WT of the second internode of the cultivars from the 2000s, 2010s, and 2020s was 42.7% (2019–2020) and 52.4% (2020–2021) higher than that of the cultivars from the 1950s–1990s (Table 2). Furthermore, the WT demonstrated an inverse correlation with N fertilizer. Compared with that at N_0_, the mean WT at N_180_ decreased by 15.8% (2019–2020) and 16.5% (2020–2021); while that at N_360_ decreased by 25.4% (2019–2020) and 23.4% (2020–2021). Similarly, the mechanical layer thickness (MLT) of the cultivars from the 2000s, 2010s, and 2020s was on average 22.7% (2019–2020) and 23.0% (2020–2021) higher than that of the others. However, the response of MLT to N fertilizer was opposite to that of the WT. Compared with that at N_0_, the mean MLT at N_180_ increased by 14.1% (2019–2020) and 11.9% (2020–2021); and that at N_360_ enhanced by 28.7% (2019–2020) and 27.8% (2020–2021).

There appeared to be no clear trend in the number of small vascular bundles (NSVB) and large vascular bundles (NLVB) among the different wheat cultivars (Table 2). However, a remarkable increase in both was observed after the addition of N. Compare to N_0_, the mean NSVB at N_180_ increased by 23.5% (2019–2020) and 20.9% (2020–2021); at N_360_ enhanced by 35.2% (2019–2020) and 31.8% (2020–2021). The mean NLVB at N_180_ increased by 7.9% (2019–2020) and 11.8% (2020–2021); at N_360_ enhanced by 10.4% (2019–2020) and 9.6% (2020–2021).

Similarly, no clear trend in the area of the SVB (ASVB) among different wheat cultivars was observed, whereas marked differences were found after the addition of N fertilizer (Table 2). Compare to that at N_0_, the mean ASVB at N_180_ increased by 21.9% (2019–2020) and 23.6% (2020–2021); that at N_360_ was enhanced by 40.7% (2019–2020) and 48.1% (2020–2021). In contrast, the mean area of LVB (ALVB) of the cultivars from the 2000s, 2010s, and 2020s was 32.6% (2019–2020) and 21.1% (2020–2021) higher than that of the cultivars from the 1950s–1990s. Compared to N_0_, the mean ALVB at N_180_ decreased by 5.8% (2019–2020) and 4.6% (2020–2021); and at N_360_ was reduced by 9.7% (2019–2020) and 10.6% (2020–2021).

### Relationship between the agronomic traits, the microstructure, and mineral elements of the stem

**Fig. 6:**
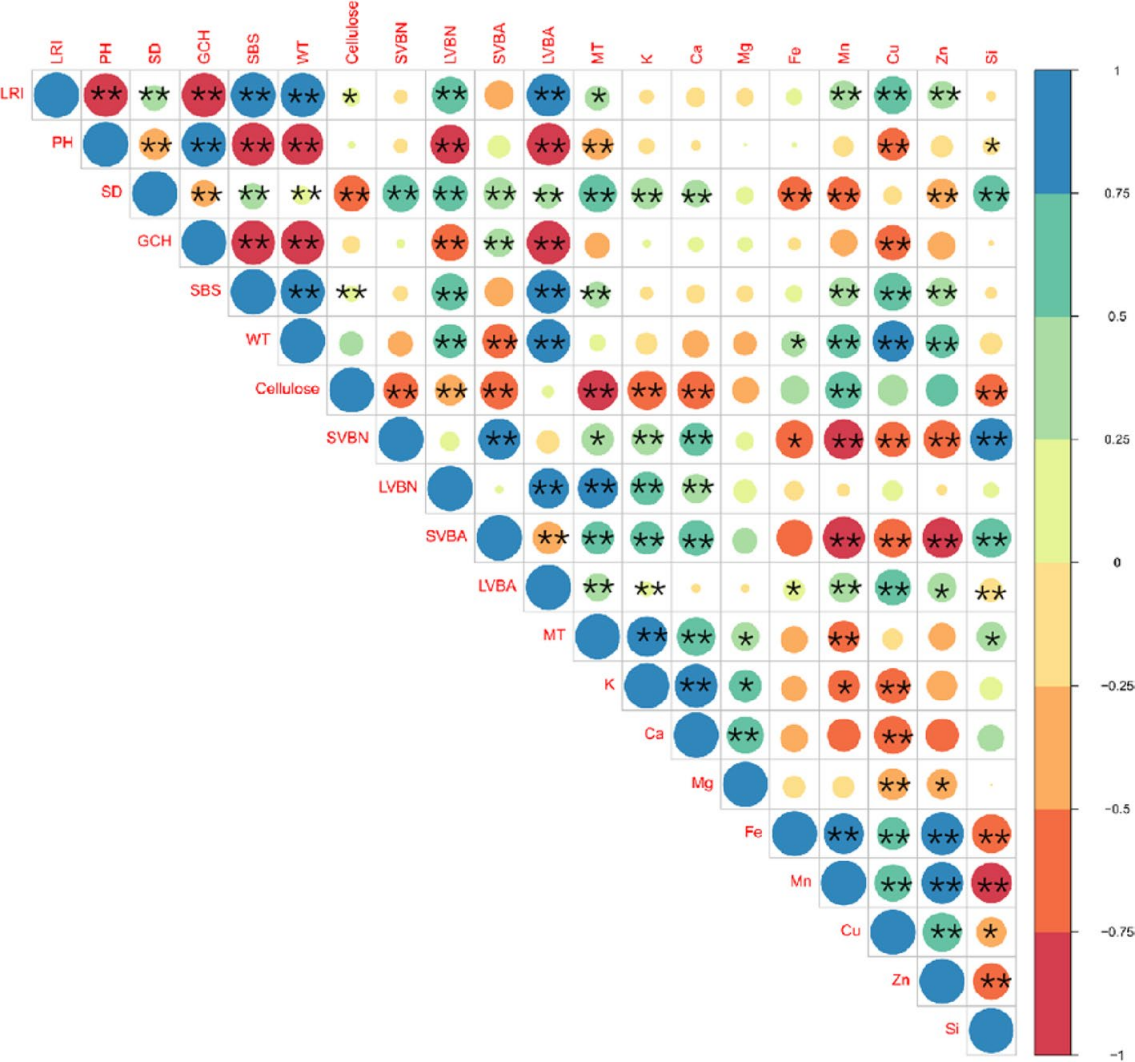
The correlation between the three N regimens and the eight varieties of wheat on the lodging resistance index and related parameters in stems. LRI, lodging resistance index; PH, plant height; SD, Stem diameter; PGC, the plant center of gravity; SBS, stem breaking strength; WT, Wall thickness; Cellulose content, CC; NSVB, number of small vascular bundles; NLBV, number of large vascular bundles; ASVB, area of small vascular bundles; ALBV, area of large vascular bundles; MTT, Mechanical tissue thickness. The chemical symbols represent the mineral elements occurring in the second internodes of the stems. * and ** indicate significant differences at the levels of 0.05 and 0.01, respectively

Figure 6 illustrates the correlation between the lodging-related traits, stem microstructure, and mineral elements. LRI was significantly correlated in a positive manner with two agronomic traits (SD and SBS), four microstructure parameters (NLVB, ALVB, WT, and mechanical tissue thickness [MTT]), and three mineral elements (Mn, Cu, and Zn); but it was significantly negatively correlated with PH, PCG, and CC. Compared to the SVBs, the LVBs were significantly correlated with more agronomic traits and mineral element content, and the AVB was more significantly correlated with more parameters in comparison with the NVB. The elements- Mn, Cu, and Zn had a significant correlation with several agronomical and microstructure traits; while K, Ca, Fe, and Si had a significant correlation with only a handful of traits; and Mg had the least correlation with any of the traits.

## Discussion

### Physiological mechanisms underlying the genetic improvement of wheat for enhancing the LR

Breeders have used dwarf and semi-dwarf phenotype-related genes to cultivate dwarf varieties, with a significantly reduced PH, improved harvest index, and enhanced LT; to date, 25 *Rht* genes have been identified in wheat, the use of which ultimately led to the development of varieties with higher GYs [8, 36]. The Green Revolution contributed to a sharp increase in the average yield of wheat over the past 50 years [37, 38]. The average wheat yield in China increased from 1840.5 kg ha^−1^ in 1978 to 5740.5 kg ha^−1^ in 2021, which was ~ 1.6 times the global average yield [28]. In this study, the yield increase in the cultivars from the 1990s–2020s was ~ 1.8 times higher than that of the cultivars from the 1950s–1980s. The yield increase achieved through genetic improvement was primarily attributed to the continuously improving grain production capacity (grain number and weight), which was accompanied by a decrease in the NPP (Table 1; Fig. 1). Wide adaptability and a strong capacity of resistance to abiotic and biotic stresses are the crucial reasons behind the significant increase in wheat yield in China after the 2000s; some dwarf, high-yielding, and disease-resistant varieties such as Zhou 8425B and 6VS/6AL play an important role when used as parents in a breeding program [39].

Lodging in plants is a crucial constraint for the stable yield and productivity of crops and is a problem that needs to be solved urgently for breeding superior, high-yielding varieties in wheat [40]. In this study, the PH remained stable in the varieties developed after the 2000s, however, the LRI was continuously improving (Figs. 4 and 5). The SBS of the modern varieties (1990s–2020s) was 45.7% higher than that of the older varieties (1950s–1980s) (Fig. 5) as it was the most dominant trait addressed in LRI improvement programs since the 2000s. Further, the SBS was significantly positively correlated with the SD and the anatomical characteristics. A previous study also revealed that 99% of the variation in the LR could be explained by the width of the MLT, thus confirming our results [18].

**Fig. 7:**
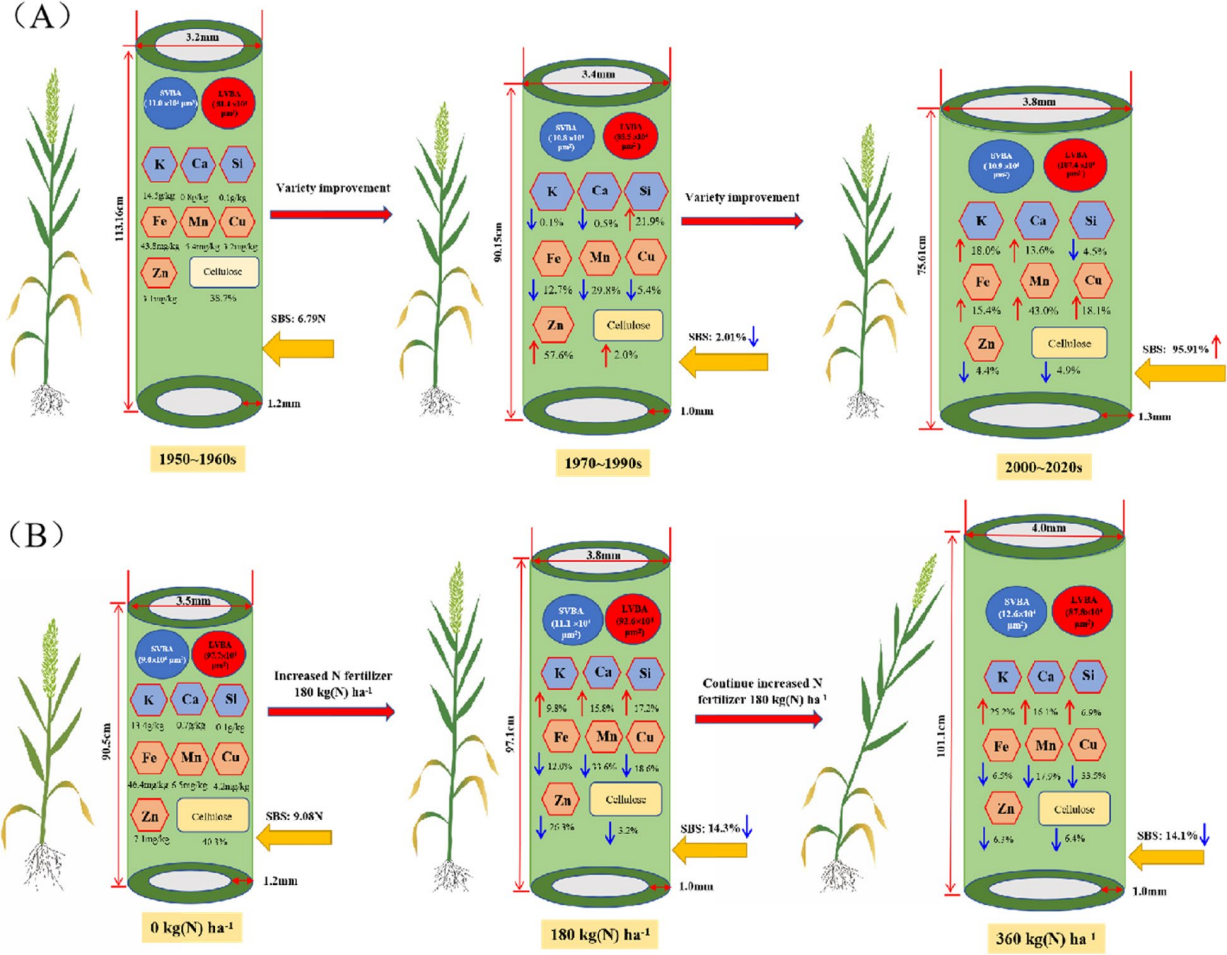
The rationale behind the lodging of wheat in the different released years (A) under different N regimens (**B**). NSVB, number of small vascular bundles; NLVB, number of large vascular bundles; ASVB, area of small vascular bundles; ALVB, area of large vascular bundles; CC, cellulose content; SBS, stem breaking strength. The chemical symbols represent the mineral elements occurring in the second internodes of the stems

The accumulation of lignin and cellulose contributed to the improvement of LR [41]. In this study, the CC of the stems was also positively correlated with the SBS. It is worth mentioning that the modern varieties had a lower CC, but a higher SBS (Table 2; Fig. 5) because the increase in stem WT compensated for the enhanced risk of lodging caused by a decrease in the CC. Certain mineral nutrients mainly K, Ca, and Si were significantly associated with stem anatomical characteristics and have been widely used in improving LR (Fig. 6). A higher K content could promote the lignification of the cells of the collenchyma and sclerenchyma and increase the accumulation of structural carbohydrates to thicken the cell wall in the stem [42]. K deficiency could cause a reduction in length, diameter, and wall thickness of culms, poor root proliferation thus increasing lodging through reduced stem strength and diameter [43]. Similarly, Ca and Si deposition in epidermal cells can also increase the cellulose and lignin contents to improve the hardness, toughness and stem elasticity [42, 44]. In addition, Mn, Cu, and Zn are also associated with the SBS, which may be due to the influence of these elements on the enhancement of the MTT, WT, and ALVB (Figs. 6 and 7). However, the mechanisms underlying the effects of these three elements on the LR have not yet been elucidated.

### Effect of N on the plasticity of LR in the different wheat varieties

The addition of N fertilizer improved the GY, but the excessive application could not further increase productivity and even negatively affected it. In this study, the application of N enhanced the yield in all the varieties and can be summarized as the 1950s–1960s > 2000s–2020s > 1970s–1990s (Fig. 1). From the perspective of yield parameters, the increased NPP was crucial in improving the yield in the older varieties, while it was both the increased NGP and NPP in the modern varieties.

Previous studies have shown that excessive application of N fertilizer increased the risk of lodging in rice [6], maize [45], and wheat [13]. The application of N led to an elevation in the levels of the endogenous GAs, which caused an elongation of the culm and hence an increase in the lodging risk [46]. In this study, it was observed that the increase in PH and the elevation in the PCG caused by N application was significantly lower in the varieties developed after the 1990s (Fig. 4). This indicates that the modern semi-dwarf varieties might be less sensitive to GA. Although the PCG increased slightly, the LRI of the modern varieties decreased significantly after N application (Fig. 3). This implies that the SBS reduced rapidly in the modern varieties after N addition. Among the various parameters that affected the SBS, the WT, the LVBA, and the CC exhibited a stronger sensitivity to N in the modern varieties. Crops with high soluble sugar content in culms are able to recover more easily from damage by strong winds and its remobilization keeps the plant alive for a longer duration during stress conditions [47, 48]. Excessive N input increased the content of soluble sugars in the leaves. However, the stems could not fully utilize these substrate to synthesize sufficient amounts of the structural carbohydrates lignin and cellulose [33]. On a molecular level, high application of N fertilizer down-regulates the expression of the genes involved in lignin and cellulose biosyntheses, causing fiber deficiency in the secondary cell walls and the weakening of mechanical tissue structure [49]. This phenomenon might be more pronounced in the modern varieties. Simultaneously, in this study, it was found that although the application of N induced the accumulation of K, Ca, and Si, the SBS significantly decreased (Fig. 5; Fig. 7). Numerous studies have confirmed that K, Ca, and Si can enhance the SBS [50, 51]. It can be concluded that the effects of mineral elements on the improvement of LR were lesser than that on the stem structure. In summary, two different types of wheat varieties are needed for the breeding-based improvement of LR in the future, the first variety with a stem anatomical structure insensitive to N fertilizer and the second variety with a higher capacity for the utilization of monosaccharides in the synthesis of cellulose and lignin polysaccharides.

## Conclusion

For the improvement of the LR, the first stage of breeding mainly depended on a decrease in the PH and an increase in the contents of cellulose, Si, and Zn; the second stage mainly depended on a sharp increase in the SBS, due to enhanced SD, stem WT, K, Ca, Fe, Mn, and Cu levels (Fig. 7A). The application of N enhanced the concentrations of the three-lodging resistance-related elements K, Ca, and Si but the LR was reduced, which was mainly due to an increase in the PH and a reduction in the LVBA and CC (Fig. 7B). This study points out a direction for improving the traits associated with increased LR through breeding and the cultivation of high-yield varieties in the future.

## Methods

### Experimental site

**Fig. 8:**
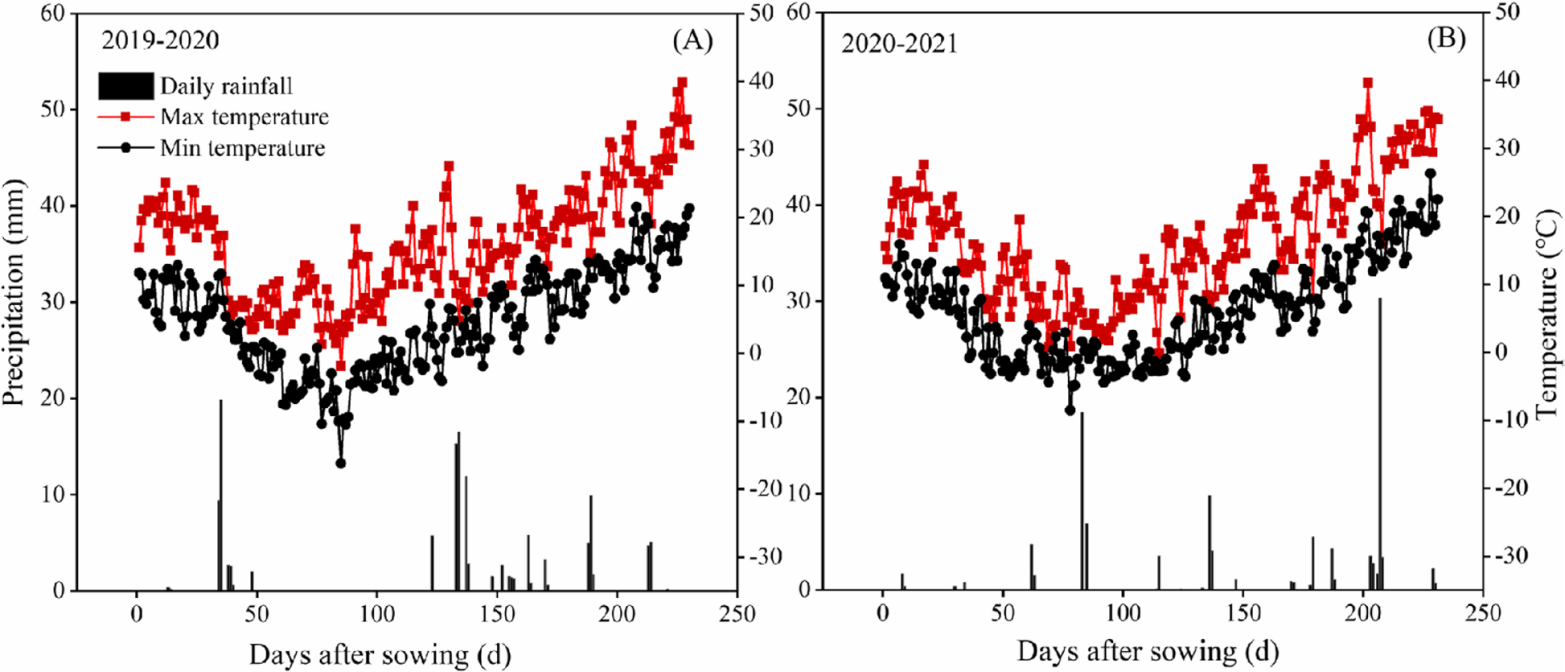
Temperature and precipitation values during the experimental seasons of 2019–2020 (**A**) and 2020–2021 (**B**)

Field experiments were conducted at the scientific research site of Henan Agricultural University (35°18′N, 113°95′E), Xinxiang County, Henan Province, China during the winter wheat cultivation season lasting from October 2019 to June 2021. During 2019–2020, the mean annual temperature was 12.1 °C, the mean winter temperature was 3.6 °C, and the total annual rainfall was 111.5 mm. During 2020–2021, the mean annual temperature was 11.2 °C, the mean winter temperature was 2.9 °C, and the total annual rainfall was 135.4 mm (Fig. 8). The soil texture was sandy, pH was 6.8, the organic matter content was 13.36 g kg^−1^ (top 0–20 cm), the total N was 1.23 g kg^−1^, the available K was 88.95 mg kg^−1^, the available P was 20.2 mg kg^−1^, and the bulk density was 1.34 g cm^−3^.

### Experimental design

**Fig. 9:**
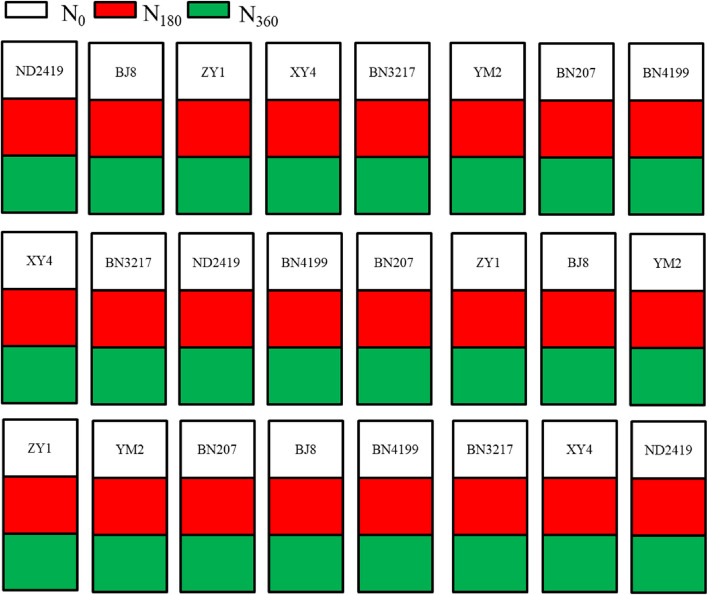
Field layout of the split-plot design experiment

Eight popular cultivars of wheat developed from the 1950s to the 2010s in the plains of North China were selected. These included Nanda 2419 (ND2419), Beijing 8 (BJ8), Zhengyin 1 (ZY1), Xiaoyan 4 (XY4), Bainong 3217 (BN3217), Yumai 2 (YM2), Bainong 207 (BN207), and Bainong 4199 (BN4199) (Table 3). The experimental design included split plots in three replications, with the dimensions of each plot being 6.0 m × 8.0 m. A total of 187.5 kg seeds ha^−1^ were mechanically sown on October 15^th^, 2019, and 2020. The plants were grown in the main plots, and the three N regimens- 0 (no N fertilizer application), 180 (recommed rate for wheat in Henan province), and 360 kg N ha^−1^ (farmer practise fertilization) were applied to the subplots, with the yield performance of the different cultivars in the field determined at the grain filling stage (Fig. 9). N (as urea) was applied at two intervals, with 50% at the basal and 50% at the jointing stage (about 160 days after sowing; DAS). P (90 kg P_2_O_5_ ha^−1^) and K (90 kg KCl ha^−1^) were applied as the basal dose. The plants were harvested on June 1^st^, 2020, and June 3^rd^, 2021. Weeds, diseases, and insects were intensively controlled during the entire growing season to avoid yield loss.

**Table 3:** The wheat cultivars used in this study. The recommended nitrogen dosage is based on yield and nitrogen application, and fitted through a linear plus plateau equation

Variety	Pedigree/Origin	Duration	Year of release	Recommended N rate (kg ha^−1^)
ND2419	Rieti × Wilhelmina∥Akagomughi	Mid-late maturing, about 229d	1950s	187.0
BJ8	BM4 × Early Premium	Mid-early maturing, about 226d	1960s	166.4
ZY1	St1472/506	Mid-late maturity, about 230d	1970s	120.1
XY4	ZY4 × ZZ17 × 6609	Mid-late maturing, about 229d	1980s	199.4
BN3217	Funo × NX5∥XN39 × XN64 × YD34	Mid-early maturity, about 226d	1990s	149.5
YM2	ZN16 × YM14	Medium maturity, about 227d	2000s	152.0
BN207	Z16 × BN64	Mid-late maturity, about 231d	2010s	137.1
BN4199	BNGG3709F2 × BNAK58	Mid-early maturity, about 226.5d	2020s	160.0

### Plant sampling and measurement of parameters

#### GY and yield parameters

At maturity, 6 m^2^ plants for each plot were harvested by manual, and then treshed by machine. The yield of wheat grains was adjusted to 13% moisture content. To determine the grain number per spike, twenty spikes from each plot were randomly selected. The 1000-grain weight was determined after the grains were dried to a constant weight at 80 ℃ in a forced ventilation oven.

#### PH and plant center of gravity (PCG)

The PH was measured from the base to the tip of the highest panicle. The center of gravity of a plant was defined as the distance from the base to the equilibrium pivot point of an entire plant with spikes, leaves, and sheaths intact [13].

#### Lodging rate and lodging resistance index

The lodging period and lodging area during the growth period were observed and recorded post-lodging. The culm characteristics related to stem lodging were determined at the grain-filling stage. The second internode from the base of the freshly cut stem was collected, the leaf sheath was stripped, and the stem breaking strength (SBS) was determined using a YYD-1A collapse resistance meter (Zhejiang Top Cloud-Agri Technology Co. Ltd., Hangzhou, China) [33]. The LR and LRI were calculated as:

1 $$\mathrm{Lodging rate }\left(\mathrm{\%}\right)=\mathrm{actual area of lodged plants in a plot }({\mathrm{m}}^{2})/\mathrm{area of the plot }({\mathrm{m}}^{2})\times 100\mathrm{\%}$$

2 $$\mathrm{LRI}=\mathrm{SBS }(\mathrm{N}) /\mathrm{ PCG }(\mathrm{cm})$$

where LRI is the lodging resistance index, SBS is the stem-breaking strength, and PCG is the plant center of gravity [52].

#### Microstructure of the basal second internode of the stem

Three uniformly sized plants of each N-treatment group were selected at the grain-filling stage. The stem segments from the middle of the basal second internode were quickly cut with a double-sided blade and fixed in an formaldehyde-acetic acid–ethanol fixative (FAA) fixation solution (containing 70% ethanol: 5% glacial acetic acid: 3.7% formaldehyde) [53]. The images of the prepared slices were collected by the slice scanner and analyzed by using the CaseViewer slice-scanning software (3DHISTECH Kft., Budapest, Hungary). This software was used to measure the WT, tissue mechanical thickness (TMT), and the number and size of the big/small-sized vascular bundles.

#### Estimation of the cellulose content (CC) of the stems

The stem samples were collected at the grain-filling stage, and the total CC was determined according to an already-described method with slight modifications [54]. 1 g of dried straw from the second internode was transferred to a 50 mL test tube to which 5 mL of a solution containing acetic acid and nitric acid in a ratio of 1:1 was added. The tubes were kept in boiling water for 30 min. Subsequently, 30 mL of distilled water was added, the tubes were centrifuged at 4000 rpm for 10 min, and the supernatant was separated. This step was repeated two more times. The collected sediments were dried in an oven at 80 °C to reach a constant weight. Then 10 mL of a solution containing 10% H_2_SO_4_ and 0.1 mol L^−1^ of K_2_Cr_2_O_7_ was added, shaken adequately, and kept in boiling water for 15 min. The precipitate was then rinsed with 30 mL of distilled water, transferred to clean Erlenmeyer flasks (Shenzhen H&Q Biologics Co. Ltd., Guangzhou, China), and allowed to cool down for titration. Finally, 5 mL of 20% KI was titrated against 0.2 mol L^−1^ Na_2_S_2_O_3_ until the solution started to turn blue and retained the same color for 30 s. The control was without straws. The CC was calculated as follows:

3 $$\mathrm{CC}=\mathrm{Concentration of }{\mathrm{Na}}_{2}{\mathrm{S}}_{2}{\mathrm{O}}_{3} ({\mathrm{mol L}}^{-1})\cdot (\mathrm{V}-{\mathrm{V}}_{0})/({\mathrm{W}}_{\mathrm{straw}}\times 24)$$

where V is the volume of Na_2_S_2_O_3_ (mL) consumed by titration with the control, V_0_ is the volume of Na_2_S_2_O_3_ (mL) consumed by titration with the test sample containing straw. W_straw_ is the dry weight of the straw (g).

#### Estimation of the levels of mineral nutrients in the stems

The plant samples were collected at the grain-filling stage, the stems were stripped of the leaf sheaths, dried at 70 °C, and then crushed to a powder. The levels of the mineral nutrients- K, Ca, Mg, Fe, Mn, Cu, Zn, and Si in the stems were determined using the MARS6 microwave digestion instrument (CEM Corporation, North Carolina, USA) and a Prodigy7 inductively coupled plasma spectrometer (Teledyne Leeman Labs, Ohio, USA).

#### Data analysis

Data were analyzed using the Statistical Software Package for Social Science (SPSS, ver 19.0) (IBM, New York, USA) and MS Office Excel 2010, the variance of analysis and mean value (*n* = 3) were compared using Fisher’s F-protected least significant difference (LSD) test at a *P* < 0.05. The graphs were plotted using the Origin 9.0 software (OriginLab Corporation, Massachusetts, USA).

## Data Availability

The datasets used and/or analysed during the current study available from the corresponding author on reasonable request.

## References

[1] Nakka S, Jugulam M, Peterson D, Asif M (2019). Herbicide resistance: development of wheat production systems and current status of resistant weeds in wheat cropping systems. Crop J.

[2] Chauhan S, Darvishzadeh R, Boschetti M, Pepe M, Nelson A (2019). Remote sensing-based crop lodging assessment: Current status and perspectives. ISPRS J Photogramm.

[3] Acreche MM, Slafer GA (2011). Lodging yield penalties as affected by breeding in Mediterranean wheats. Field Crop Res.

[4] Berry PM, Berry ST (2015). Understanding the genetic control of lodging-associated plant characters in winter wheat (Triticum aestivum L.). Euphytica.

[5] Paulo F, Zhao Z (2021). Wheat lodging ratio detection based on UAS imagery coupled with different machine learning and deep learning algorithms. Smart Agr.

[6] Zhang Y, Xu W, Wang H, Fang Y, Dong H, Qi X (2016). Progress in improving stem lodging resistance of Chinese wheat cultivars. Euphytica.

[7] Wilhelm EP, Boulton MI, Barber TES, Greenland AJ, Powell W (2013). Genotype analysis of the wheat semi-dwarf Rht-B1b and Rht-D1b ancestral lineage. Plant Breed.

[8] McIntosh RA, Yamazaki Y, Dubcovsky J, Rogers J, Morris C, Appels R Xia XC. Catalogue of gene symbols for wheat: 2020 supplement. In: 12th Int Wheat Genet Symp. 2013.

[9] Zhao K, Xiao J, Liu Y, Chen S, Yuan C, Cao A, You FM, Yang D, An S, Wang H (2018). Rht23 (5Dq’) likely encodes a Q homeologue with pleiotropic effects on plant height and spike compactness. Theor Appl Genet.

[10] Mo Y, Vanzetti LS, Hale I, Spagnolo EJ, Guidobaldi F, Al-Oboudi J, Odle N, Pearce S, Helguera M, Dubcovsky J (2018). Identification and characterization of Rht25, a locus on chromosome arm 6AS affecting wheat plant height, heading time, and spike development. Theor Appl Genet.

[11] Tian X, Wen W, Xie L, Fu L, Xu D, Fu C, Wang D, Chen X, Xia X, Chen Q (2017). Molecular mapping of reduced plant height gene Rht24 in bread wheat. Front Plant Sci.

[12] Niu Y, Chen T, Zhao C, Guo C, Zhou M. Identification of QTL for stem traits in wheat (Triticum aestivum L.). Front. Plant Sci. 2022;13:962253. 10.3389/fpls.2022.962253 PMC9330363 35909739

[13] Kong E, Liu D, Guo X, Yang W, Sun J, Li X, Zhan K, Cui D, Lin J, Zhang A (2013). Anatomical and chemical characteristics associated with lodging resistance in wheat. Crop J.

[14] Pinera-Chavez FJ, Berry PM, Foulkes MJ, Sukumaran S, Reynolds MP (2021). Identifying quantitative trait loci for lodging-associated traits in the wheat doubled-haploid population Avalon × Cadenza. Crop Sci.

[15] Zhou CY, Xiong HC, Li YT, Guo HJ, Xie YD, Zhao LS, Gu JY, Zhao SR, Ding YP, Song XY, Liu LX (2020). Genetic analysis and QTL mapping of a novel reduced height gene in common wheat (Triticum aestivum L.). J Integr Agric..

[16] Tang T, Acuna Botwright, Spielmeyer W, Richards RA (2021). Effect of gibberellin-sensitive Rht18 and gibberellin-insensitive Rht-D1b dwarfing genes on vegetative and reproductive growth in bread wheat. J Exp Biol.

[17] Austin RB, Bingham J, Blackwell RD, Evans LT, Ford MA, Morgan CL, Taylor M (1980). Genetic improvements in winter wheat yields since 1900 and associated physiological changes. J Agr Sci.

[18] Zhang HJ, Li T, Liu HW, Mai CY, Yu GJ, Li HL, Yu LQ, Meng LZ, Jian DW, Yang L, Li HJ, Zhou Y (2020). Genetic progress in stem lodging resistance of the dominant wheatcultivars adapted to Yellow-Huai river valleys winter wheat zonein China since 1964. J Integr Agr.

[19] Wu W, Ma BL, Fan JJ, Sun M, Yi Y, Guo WS, Voldeng HD (2019). Management of nitrogen fertilization to balance reducing lodging risk and increasing yield and protein content in spring wheat. Field Crop R.

[20] Zheng MJ, Chen J, Shi YH, Li YX, Yin YP, Yang DQ, Luo YL, Pang DW, Li WQ, Ni J, Wang YY, Wang ZL, Li Y (2017). Manipulation of lignin metabolism by plant densities and its relationship with lodging resistance in wheat. Sci Rep.

[21] Kamran M, Ahmad I, Wang HQ, Wu XR, Xu J, Li TN, Ding RX, Han QF (2018). Mepiquat chloride application increases lodging resistance of maize by enhancing stem physical strength and lignin biosynthesis. Field Crop Res.

[22] Kamran M, Cui W, Ahmad I, Meng XP, Zhang X, Su W, Chen J, Ahmad S, Fahad S, Han QF, Liu T. Effect of paclobutrazol, a potential growth regulator on stalk mechanical strength, lignin accumulation and its relation with lodging resistance of maize. Plant Growth Regul. 2018;84:317–32.

[23] Kamran M, Ahamd I, Wu XR, Liu TN, Ding RX, Han  QF (2018). Application of paclobutrazol: a strategy for inducing lodging resistance of wheat through mediation of plant height, stem physical strength, and lignin biosynthesis</div>. Environ Sci Pollut R.

[24] Caffall KH, Mohnen D (2009). The structure, function, and biosynthesis of plant cell wall pectic polysaccharides. Carbohydr Res.

[25] Reddy N, Yang Y (2005). Structure and properties of high quality natural cellulose fibers from cornstalks. Polymer.

[26] Jinger D, Dhar S, Dass A, Sharma VK, Kumar V, Gupta G (2020). Influence of residual silicon and phosphorus on growth, productivity, lodging and grain quality of succeeding wheat under rice-wheat cropping system. J Environ Biol.

[27] Tarigan DM, Syofia I, Barus WA, Munar A (2019). Lodging characters of wheat: Effect of nitrogen and potassium combination. Int J Sci Tech R.

[28] Xiao J, Liu B, Yao YY, Guo ZF (2022). Wheat genomic study for genetic improvement of traits in China. Sci China.

[29] Zhang GX, Liu SJ, Dong YJ, Liao YC, Han J (2022). A nitrogen fertilizer strategy for simultaneously increasing wheat grain yield and protein content: Mixed application of controlled-release urea and normal urea. Field Crop R.

[30] Zheng BQ, Jiang JL, Wang LL, Huang M, Zhou Q, Cai J, Wang X, Dai TB, Jiang D (2022). Reducing nitrogen rate and increasing plant density accomplished high yields with satisfied grain quality of soft wheat via modifying the free amino acid supply and storage protein gene expression. J Agr Food Chem.

[31] Wu W, Ma BL (2018). Assessment of canola crop lodging under elevated temperatures for adaptation to climate change. Agric Forest Meteorol.

[32] Sun Q, Liu XG, Yang J, Liu WW, Du QG, Wang HQ, Fu CX, L WX. MicroRNA528 affects lodging resistance of maize by regulating lignin biosynthesis under nitrogen-luxury conditions. Mol Plant. 2018;11:806–14. 10.1016/j.molp.2018.03.013 29597009

[33] Li CH, Chang YL, Luo YL, Li WQ, Jin M, Wang YY, Cui HX, Sun SF, Li Y, Wang ZL (2023). Nitrogen regulates stem lodging resistance by breaking the balance of photosynthetic carbon allocation in wheat. Field Crop Res.

[34] Niu YN, Chen TX, Zhao CC, Zhou MX (2022). Lodging prevention in cereals: Morphological, biochemical, anatomical traits and their molecular mechanisms, management and breeding strategies. Field Crop Res.

[35] Pan JF, Zhao JL, Liu YZ, Huang NR, Tian K, Shah F, Liang KM, Zhong XH, Liu B (2019). Optimized nitrogen management enhances lodging resistance of rice and its morpho-anatomical, mechanical, and molecular mechanisms. Sci Rep.

[36] Gale MD, Youssefian S, Russell G (1985). Dwarfing genes in wheat. Prog Plant Breed.

[37] Gupta P (2016). Use of alien genetic variation for wheat improvement. Molecular Breeding for Sustainable Crop Improvement.

[38] Molnár-Láng M, Ceoloni C, Doležel J (2015). Alien Introgression in Wheat.

[39] He ZH, Xia XC, Chen XM, Zhuang QS (2011). Progress of wheat breeding in China and the future perspective. Acta Agr Sin.

[40] Kashiwagi T, Ishimaru K (2004). Identification and functional analysis of a locus for improvement of lodging resistance in rice. Plant Physiolo.

[41] Irshad A, Meng XP, Muhammad K, Shahzad A, Shakeel A, Liu TN, Cai T, Hang QF (2020). J Inter Agr.

[42] Zhang F, Jin Z, Ma G, Shang W, Liu H, Xu M, Liu Y (2010). Relationship between lodging resistance and chemical contents in culms and sheaths of japonica rice during grain filling. Rice Sci.

[43] Crook MJ, Ennos AR (1995). The effect of nitrogen and growth regulators on stem and root characteristics associated with lodging in two cultivars of winter wheat. J Exp Bot.

[44] Thomas CB, Soren H, Kristian HL, Daniel PP, Jan KS (2021). The molecular–physiological functions of mineral macronutrients and their consequences for deficiency symptoms in plants. New Phytol.

[45] Liu XM, Gu WR, Li CF, Li J, Wei S (2021). Effects of nitrogen fertilizer and chemical regulation on spring maize lodging characteristics, grain filling and yield formation under high planting density in Heilongjiang province China. J Inter Agr.

[46] Wang Y, Ren T, Lu JW, Cong RH, Hou WF, Liu T, Saddam H, Li XK (2017). Exogenously applied gibberellic acid improves the growth and yield performance of inferior rice tillers grown under different nitrogen levels. Acta Physiol Plant.

[47] Ishimaru K, Yano M, Aoki N, Ono K, Hirose T, Lin SY, Monna L, Sasaki T, Ohsugi R (2001). Toward the mapping of physiological and agronomic characters on a rice function map: QTL analysis and comparison between QTLs and expressed sequence tags. Theor Appl Genet.

[48] Thomas H, Howarth CJ (2000). Five ways to stay green. J Exp Bot.

[49] Zhang W, Wu L, Ding Y, Yao X, Wu X, Weng F, Li G, Liu Z, Tang S, Ding C (2017). Nitrogen fertilizer application affects lodging resistance by altering secondary cell wall synthesis in japonica rice (Oryza sativa). J Plant Res.

[50] Zhang TT, He XP, Chen BL, Tang XR (2021). Effects of different potassium fertilizer rates on yield formation and lodging of rice. Phyton-Int J Exp Bot.

[51] Bhagat KP, Sairam RK, Deshmukh PS (2011). Biochemical analysis of stem in lodging tolerant and susceptible wheat (Triticum aestivum L.) genotypes under normal and late sown conditions. Indian J Plant Physiol..

[52] Xiang DB, Zhao G, Wan Y, Tan ML, Song C, Song Y (2016). Effect of planting density on lodging-related morphology, lodging rate, and yield of tartary buckwheat (Fagopyrum tataricum). Plant Pord Sci.

[53] Zhang WJ, Wu LW, Wu XR, Ding YF, Li GH, Li JY, Weng F, Liu ZH, Tang S, Ding CQ, Wang SH (2016). Lodging resistance of Japonica rice (oryza sativa L.): morphological and anatomical traits due to top-dressing nitrogen application rates. Rice.

[54] Lavanya M, Mauricio U, Paxton P, Cecilia M, Jennifer C, Venugopal M (2022). Lignin and cellulose content differences in roots of different cotton cultivars associated with different levels of Fusarium wilt race 4 (FOV4) resistance-response. J Agr Food Res.

